# Supplementary material for: Anti-Inflammatory Activity and Mechanism of Isookanin, Isolated by Bioassay-Guided Fractionation from Bidens pilosa L
Source: Molecules. 2021 Jan 6;26(2):255. doi: 10.3390/molecules26020255 (PMC7825412; doi:10.3390/molecules26020255)

SUPPLEMENTARY MATERIAL

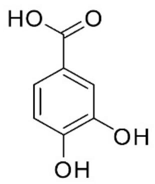

1

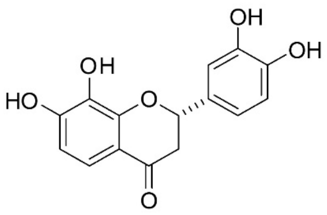

2

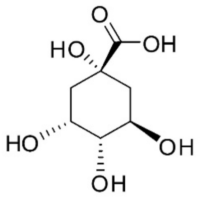

3

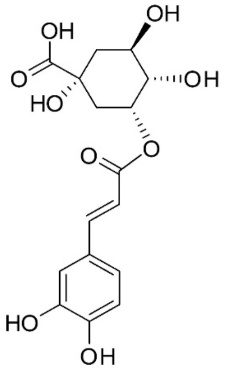

4

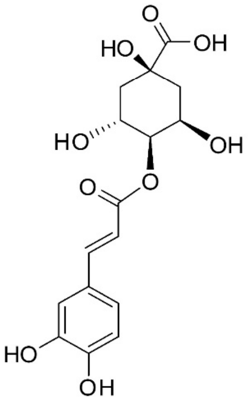

5

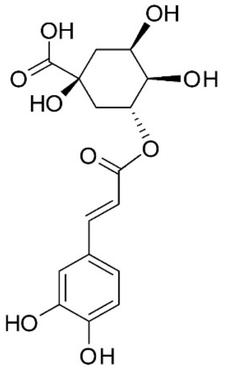

6

Figure S1. Chemical structures of compounds (1-6)

A

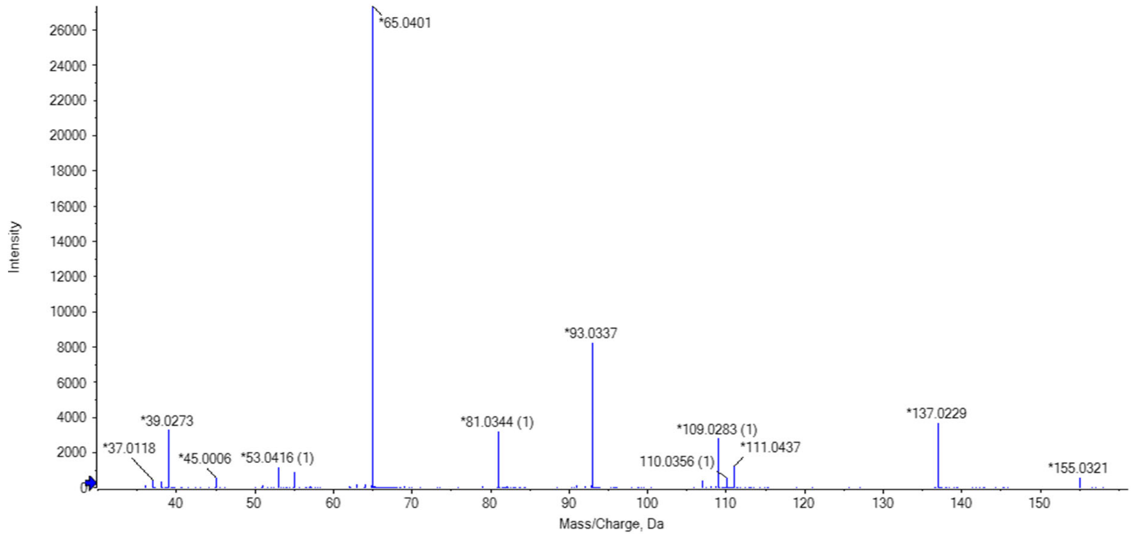

B

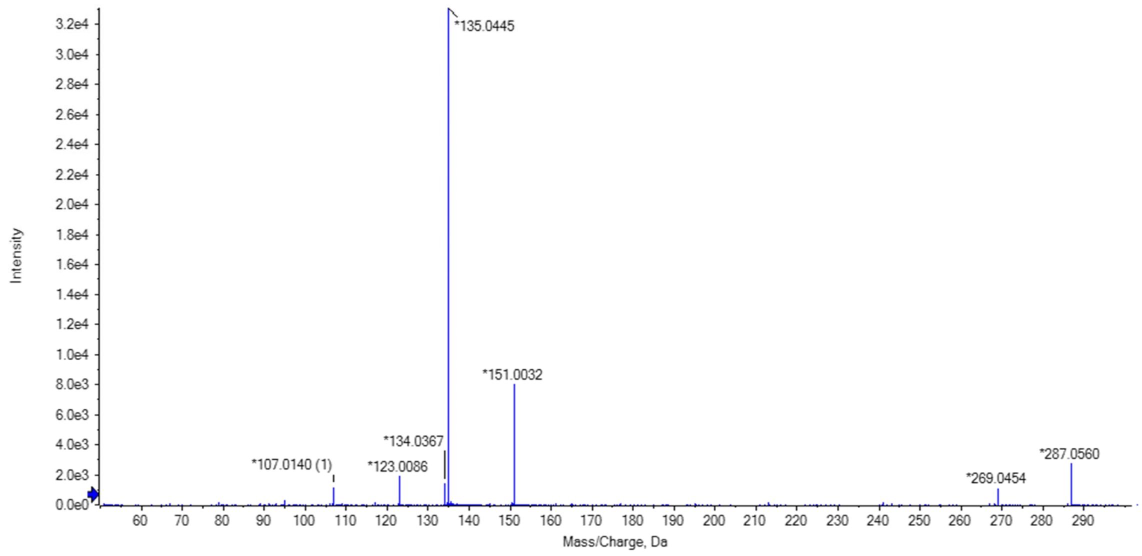

Figure S2. HR-ESI MS/MS spectra of compound **1** (A. protocatechuic acid) and compound **2** (B. isookanin).

A

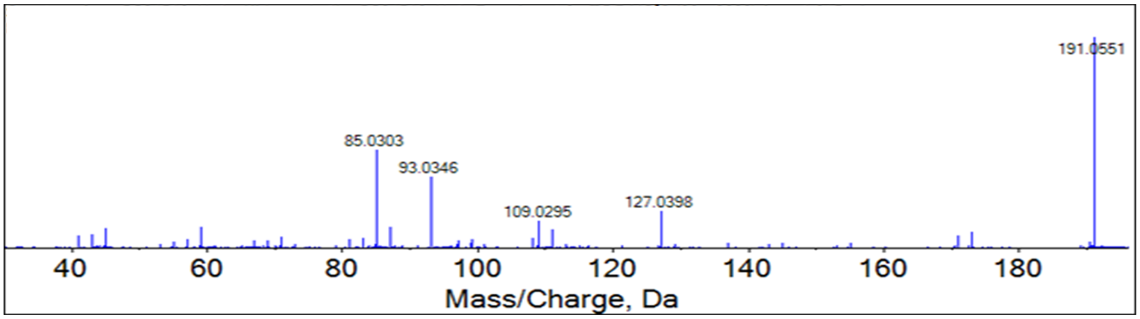

B

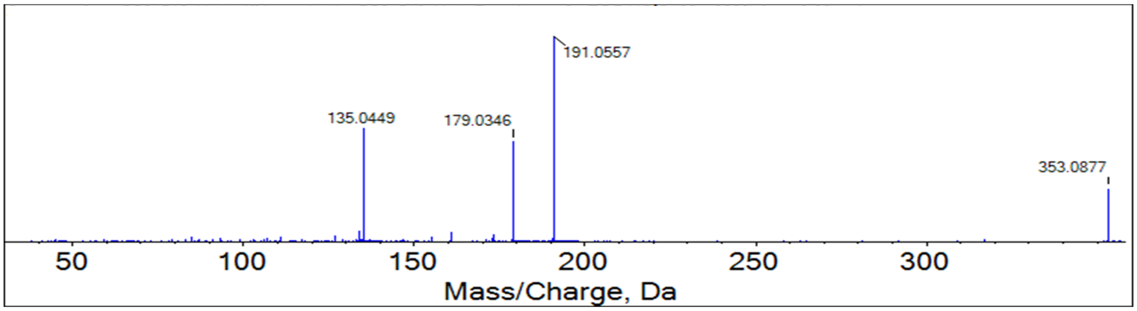

C

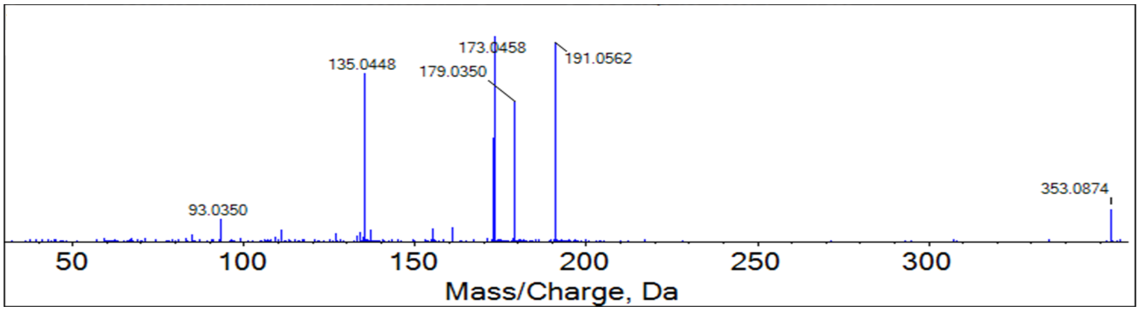

D

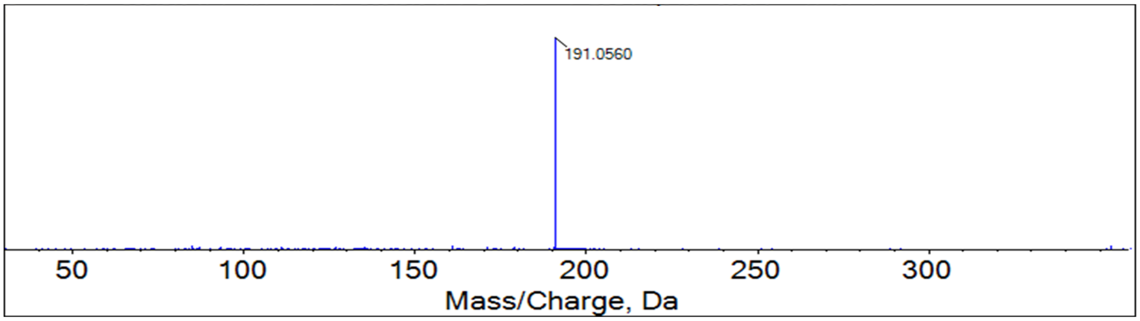

Figure S3. HR-ESI MS/MS spectra of compound **3** (A. (-)-quinic acid), compound **4** (B. 3-*O*-caffeoylquinic acid), compound **5** (C. 4-*O*-caffeoylquinic acid) and compound **6** (D. 5-*O*-caffeoylquinic acid).

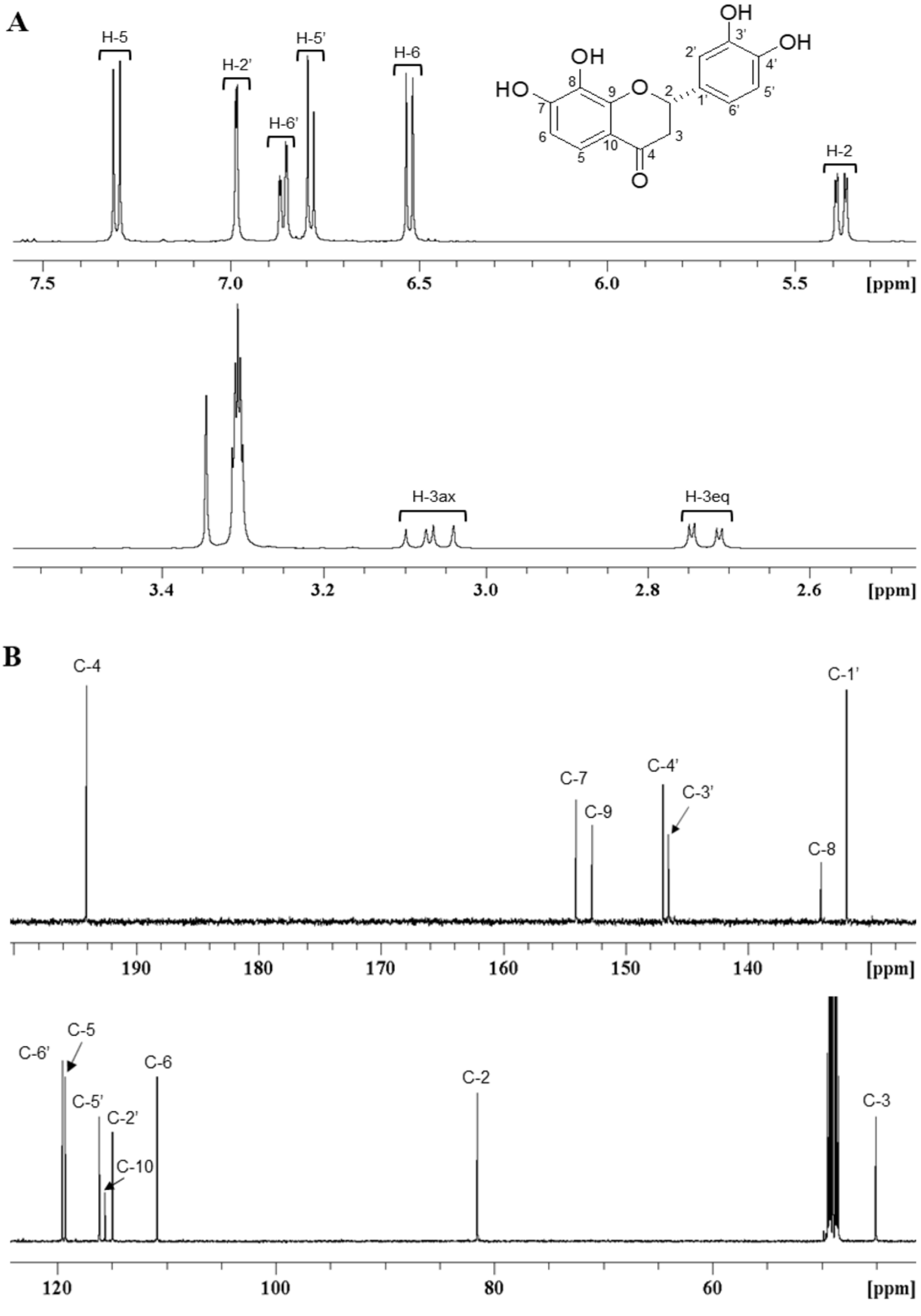

Figure S4. 1D NMR spectra of isookanin (**2**). (A) ^{1}H NMR spectrum and (B) ^{13}C NMR spectrum. Inset shows the chemical structure of isookanin.

Full length Western blot

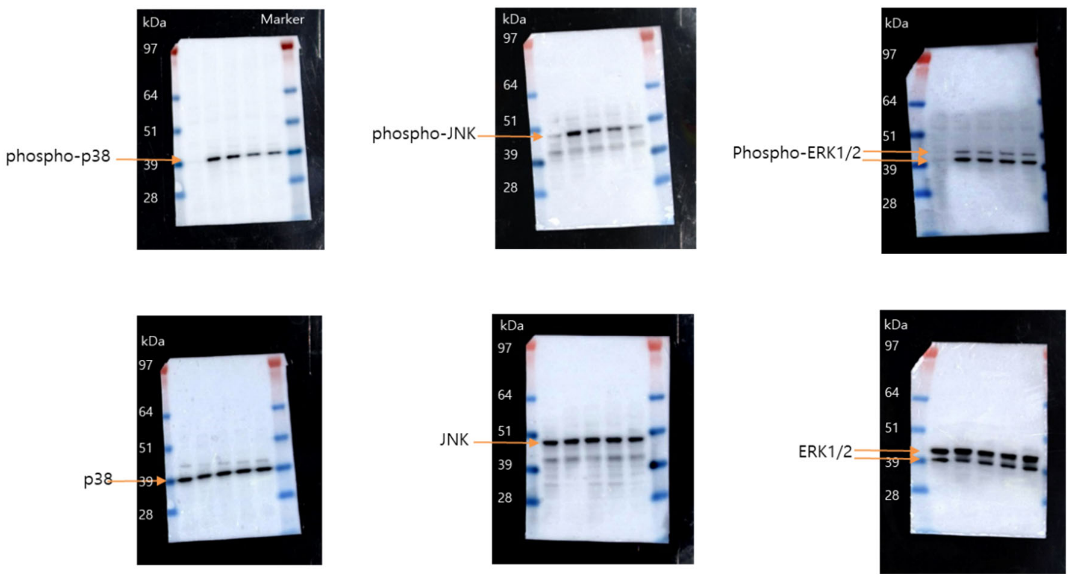

Supplement: Supplementary file 1 [file molecules-26-00255-s001.pdf]
